# Risk factors of pelvic floor muscle strength in south Chinese women: a retrospective study

**DOI:** 10.1186/s12884-022-04952-0

**Published:** 2022-08-06

**Authors:** Jianqi Fang, Jiajia Ye, Qing Huang, Yang Lin, Yilin Weng, Miao Wang, Yi Chen, Yao Lu, Ronghua Zhang

**Affiliations:** 1 Department of Women’s Health Care, Fujian Maternity and Child Health Hospital, Fuzhou, Fujian 350000 People’s Republic of China; 2 Department of Rehabilitation Assessment, Rehabilitation Hospital Affiliated to Fujian University of Traditional Chinese Medicine, Fuzhou, Fujian 350000 People’s Republic of China; 3 Department of Rehabilitation Assessment, Rehabilitation Hospital, Fujian University of Traditional Chinese Medicine, Fujian 350000 Fuzhou, People’s Republic of China; 4 College of Environment and Public Health, Xiamen Huaxia University, Xiamen Fujian, People’s Republic of China

**Keywords:** Surface electromyography, Postpartum, Pelvic floor muscle strength, Risk factors, South China

## Abstract

**Objectives:**

To evaluate pelvic floor muscle strength using surface electromyography and risk factors for pelvic floor muscle strength in the early postpartum period.

**Methods:**

This retrospective study included 21,302 participants who visited Fujian Maternity and Child Health Hospital from September 2019 to February 2022. All participants were assessed by medical professionals for general information and surface electromyography.

**Results:**

Univariate analysis indicated that age was inversely related to tonic and endurance contractions. In contrast, all the other variables, including education level, body mass index, neonatal weight, and number of fetuses, had a positive impact on rapid, tonic, and endurance contractions. Likewise, parity was also positively associated with rapid contractions. In addition, compared with vaginal delivery, cesarean section delivery had a protective effect on the amplitude of the three types of contractions. Stepwise regression analysis showed that both age and neonatal weight had a negative linear relationship with the amplitude of rapid, tonic and endurance contractions. In contrast, the amplitude of rapid, tonic and endurance contractions significantly increased as body mass index, parity (≤ 3), education level and gestational weight gain (endurance contractions only) increased. Participants with cesarean section delivery showed positive effects on rapid, tonic, and endurance contractions compared to participants with vaginal delivery.

**Conclusions:**

We found that age, neonatal weight, vaginal delivery, episiotomy, and forceps delivery were risk factors for pelvic floor muscle strength; in contrast, body mass index, parity (≤ 3) and gestational weight gain had a positive relationship with pelvic floor muscle strength.

## Introduction

The pelvic floor is composed of three layers of muscles combined with ligaments and fascia that act as a sling to support the bladder, reproductive organs, and rectum [1, 2]. The pelvic floor muscles (PFMs) function to regulate the storage and evacuation of urine and stool by coordinated contraction and relaxation. In normal people, for the purpose of preventing urinary incontinence (UI), the PFMs needs to be flexible enough to contract, providing additional external support for the urethra to cope with sudden increased intra-abdominal pressure, such as the pressure induced by coughing [3]. Similarly, the contraction of the PFMs also plays an important role in anal continence function [4]. Both pregnancy and subsequent vaginal delivery may lead to levator plate relaxation and thus increase the risk of developing pelvic floor dysfunctions (PFDs), especially UI [5–8]. The prevalence of UI in adult women was approximately 22.1% in China, 16.7% in Japan, 53% in the United States, 35.3% in Australia, and 36.3% in Saudi countries, and the prevalence of fecal incontinence (FI) was 8.39% in America, 3.6% in the United Kingdom, and 4.2% in Italy based on large population-studies [9–15]. PFDs are serious problems that can decrease participation in sports and social functions, and that have an indisputable impact on quality of life [16, 17]. With increasing age, the volume of PFMs decreases, the strength of PFMs weakens, and the incidence of PFDs increases [18, 19]. In contrast, stronger PFM strength has a great protective effect on the pelvic floor and reduces the occurrence of PFDs [20, 21].

Therefore, it is important to assess PFM strength and determine the factors that may affect it. The aim of this study was to evaluate PFM strength using surface electromyography (EMG) and risk factors for PFM strength in the early postpartum period.

## Materials and methods

### Participants

This retrospective study included 21,302 participants who visited Fujian Maternity and Child Health Hospital from September 2019 to February 2022. All participants were assessed by medical professionals for general information and surface EMG. The inclusion criteria were as follows: participants who were 40 days to 6 months postpartum and who could tolerate a gynecological examination [22]. The exclusion criteria were: participants with gynecologic bleeding, those suspected of being pregnant, and those who had undergone urogynecological and gynecological surgeries. This study was approved by the Ethics Committee of Fujian Maternity and Child Health Hospital (No. 2022KYLLR03046).

### Assessment of pelvic floor surface EMG

In China, the assessment of EMG has been routinely used to evaluate pelvic floor conditions for years [23]. A human biostimulation feedback instrument (MLD B2T, Medlander, Najing, Jiangsu, China) was used to evaluate the EMG of the participants, including pretest resting, rapid contractions, tonic contractions, endurance contractions, and posttest resting, following the Glazer protocols [24]. The participants who underwent the test were placed in the supine lithotomy position, and then a vaginal probe was placed into the vagina. Electrode configurations were positioned on abdominal muscles to monitor unwanted muscle activation. The evaluator instructed them to perform vaginal contractions, guided by words such as "Please relax your abdomen and hips", "Please contract and relax your vagina or anus quickly" and "Please contract your vagina or anus and holding". Then, the automated protocol software instructed the participants with text hints on a screen and voice prompts. In addition, our staff also supervised participants to avoid false contractions. There was a 30-s study period before the test to ensure that the participants had mastered the test correctly.

### Statistical analysis

All statistical analyses were performed using SPSS software version 26.0. Univariable analysis for categorical and continuous parameters was performed with chi square tests and t tests, respectively. The greater the absolute value of the standardized regression coefficient (β), the greater the influence of the corresponding independent variable on the dependent variable. Stepwise regression analysis was used to assess the relationship between the independent and dependent variables. For all tests, a two-tailed p value < 0.05 was considered statistically significant.

## Results

A total of 4511 participants were excluded, and 21,302 participants were included in this analysis. The mean age, height, weight, body mass index (BMI), gestational weight gain (GWG), and neonatal weight (NW) were 30.43 ± 4.035 years, 160.37 ± 5.240 cm, 59.78 ± 8.084 kg, 23.23 ± 2.834 kg, 12.88 ± 4.651 kg, 3.27 ± 0.516 kg respectively. There were 9066 (42.6%) participants who were younger than 29 years, 11,809 (55.4%) who were aged 30–39 years, and 427 (2.0%) who were 40–49 years. A total of 590 (2.8%) participants had a BMI less than 18.5, 13,002 (61.0%) had a BMI from 18.5–23.9, 6508 (30.6%) had a BMI from 24–27.9, and 1202 (5.6%) had a BMI from more than 28. A total of 13,211 (62.0%) participants had a parity of one, 7348 (34.5%) had a parity of two, 698 (3.3%) had a parity of three, and 45 (0.2%) had a parity more than three. A total of 3834 (18.0%) participants received less than 12 years of education, and 17,468 (82.0%) received more than 12 years. A total of 20,086 (94.3%) of the infants weighed less than 4 kg, and 1216 (5.7%) weighed more than 4 kg. A total of 20,860 (97.9%) participants had single births and 442 (2.1%) had twin or triplet births. A total of 7664 (36.0%) participants had a cesarean section (CS), 10,481 (49.2%) had a noninstrumental vaginal delivery (NIVD), 2600 (12.2%) had an episiotomy (EP), and 557 (2.6%) had a forceps delivery (FD). The baseline demographic features are summarized in Table 1.

**Table 1:** General characteristics of research participants

Variables	Group	Number (%)	Mean ± SD (median)
Age	≤ 29	9066 (42.6)	30.43 ± 4.035
	30–39	11,809 (55.4)	
	40–49	427 (2.0)	
Height			160.37 ± 5.240
Weight			59.78 ± 8.084
BMI(kg/m2)	< 18.5	590 (2.8)	23.23 ± 2.834
	18.5–23.9	13,002 (61.0)	
	24–27.9	6508 (30.6)	
	≥ 28	1202 (5.6)	
Parity	1	13,211 (62.0)	
	2	7348 (34.5)	
	3	698 (3.3)	
	≥ 4	45 (0.2)	
Education	≤ 12	3834 (18.0)	
	> 12	17,468 (82.0)	
GWG			12.88 ± 4.651
NW	< 4	20,086 (94.3)	3.27 ± 0.516
	≥ 4	1216 (5.7)	
NOF	1	20,860 (97.9)	
	≥ 2	442 (2.1)	
DM	CS	7664 (36.0)	
	NIVD	10,481 (49.2)	
	EP	2600 (12.2)	
	FD	557 (2.6)	

*BMI* body mass index, *GWG* gestational weight gain, *NW* neonatal weight, *NOF* number of fetus, *DM* delivery mode, *CS* cesarean section, *NIVD* non-instrumental vaginal delivery, *EP* episiotomy, *FD* forceps delivery

Univariate analysis indicated that age was inversely related to tonic contractions and endurance contractions (*P* < 0.001, and *P* < 0.001, respectively). In contrast, all the other variables, including education level, BMI, NW, and NOF, had a positive impact on rapid contractions, tonic contractions, and endurance contractions (*P*=0.003, *P*<0.001, *P*<0.001, respectively; *P* < 0.001, *P* < 0.001, *P* < 0.001, respectively; *P* < 0.001, *P* < 0.001, *P* < 0.001, respectively; *P* < 0.001, *P* < 0.001, *P* < 0.001, respectively). Likewise, parity was also associated with rapid contractions (*P* < 0.001), and the average strength significantly increased as the number of parities increased. In addition, CS delivery also had a protective effect on PFM strength, including the three types of contractions, compared with NIVD (*P* < 0.001, *P* < 0.001, *P* < 0.001, respectively), EP (*P* < 0.001, *P* < 0.001, *P* < 0.001, respectively), and FD (*P* < 0.001, *P* < 0.001, *P* < 0.001, respectively) (Table 2).

**Table 2:** Changes in rapid, tonic, and endurance contraction according to participants general characteristics in univariate analysis

Variables	Group	Rapid contraction		Tonic contraction		Endurance contraction	
		Mean ± SD	*P*	Mean ± SD	*P*	Mean ± SD	*P*
Age	≤ 29	37.49 ± 17.70	0.420	26.38^c^ ± 13.27	< 0.001	22.22^b^ ± 11.47	< 0.001
	30–39	37.82 ± 18.64		25.75^b^ ± 13.47		21.55^a^ ± 11.48	
	40–49	37.91 ± 18.27		23.81^a^ ± 12.68		20.53^a^ ± 11.77	
BMI(kg/m2)	< 18.5^a^	32.61 ± 15.12	< 0.001	22.80 ± 11.75	< 0.001	19.26 ± 10.19	< 0.001
	18.5–23.9^b^	36.67 ± 17.68		25.31 ± 12.83		21.23 ± 11.08	
	24–27.9^c^	39.32 ± 18.60		27.06 ± 14.17		22.69 ± 11.96	
	≥ 28^d^	42.26 ± 21.70		28.95 ± 14.50		24.66 ± 12.89	
Parity	1	36.98^a^ ± 17.68	< 0.001	25.92 ± 13.38	0.293	21.79 ± 11.52	0.445
	2	38.83^b^ ± 19.05		26.12 ± 13.35		21.85 ± 11.41	
	3	39.02^b^ ± 19.48		25.90 ± 13.84		22.09 ± 11.80	
	≥ 4	33.63 ± 13.54		22.717 ± 9.67		19.32 ± 8.27	
Education	≤ 12	36.89 ± 18.66	0.003	24.45 ± 12.93	< 0.001	20.79 ± 11.08	< 0.001
	> 12	37.85 ± 18.14		26.32 ± 13.45		22.04 ± 11.56	
NW	< 4	37.51 ± 18.09	< 0.001	25.86 ± 13.34	< 0.001	21.69 ± 11.44	< 0.001
	≥ 4	40.43 ± 20.36		28.04 ± 13.87		23.93 ± 12.04	
NOF	1	37.57 ± 18.21	< 0.001	25.90 ± 13.35	< 0.001	21.74 ± 11.46	< 0.001
	≥ 2	42.74 ± 19.06		29.96 ± 14.22		25.47 ± 12.17	
DM	CS^d^	44.74 ± 18.88	< 0.001	31.23 ± 14.01	< 0.001	26.61 ± 12.09	< 0.001
	NIVD^c^	34.49 ± 16.85		23.53 ± 12.11		19.55 ± 10.25	
	EP^b^	32.58 ± 15.49		22.48 ± 11.61		18.58 ± 9.84	
	FD^a^	24.29 ± 13.29		16.28 ± 10.44		13.50 ± 8.71	

Post-hoc test: a < b < c < d

*BMI* body mass index, *NW* neonatal weight, *NOF* number of fetus, *DM* delivery mode, *CS* cesarean section, *NIVD* non-instrumental vaginal delivery, *EP* episiotomy, *FD* forceps delivery

Stepwise regression analysis showed that age and NW had a negative linear relationship with rapid, tonic and endurance contractions (β = -0.066, *P* < 0.001; β = -0.107, *P* < 0.001; β = -0.109, *P* < 0.001, respectively; β = -0.034, *P* < 0.001; β = -0.015, *P* < 0.05; β = -0.020, *P* < 0.01, respectively). Secundiparas showed a positive effect on rapid, tonic, and endurance contractions compared with primiparas (β = -0.055, *P* < 0.001; β = -0.032, *P* < 0.001; β = -0.029, *P* < 0.001, respectively). All factors showed even positive values for tertiparas (β = -0.025, *P* < 0.001; β = -0.018, *P* < 0.05; β = -0.021, *P* < 0.01, respectively). In contrast, BMI, education level and GWG (endurance contractions only) also showed a positive linear relationship with three types of contractions (β = 0.085, *P* < 0.001; β = 0.078, *P* < 0.001; β = 0.076, *P* < 0.001, respectively; β = 0.058, *P* < 0.001; β = 0.090, *P* < 0.001; β = 0.080, *P* < 0.001, respectively; β = 0.019, *P* < 0.01). Participants with CS delivery showed a positive effect on rapid, tonic, and endurance contractions compared with participants with NIVD (β = -0.292, *P* < 0.001; β = -0.305, *P* < 0.001; β = -0.324, *P* < 0.001, respectively), EP (β = -0.216, *P* < 0.001; β = -0.224, *P* < 0.001; β = -0.239, *P* < 0.001, respectively), and FD (β = -0.176, *P* < 0.001; β = -0.182, *P* < 0.001; β = -0.185, *P* < 0.001, respectively). (Table 3).

**Table 3:** Stepwise multiple linear regression analysis for the effect of independent variables on rapid, tonic, and endurance contraction

Variables		Rapid contraction				Tonic contraction				Endurance contraction		
		B	β	t		B	β	t		B	β	t
(constant)		38.668		24.032		28.967		24.350		25.298		24.770
Age		-.298	-.066	-9.123^***^		-.354	-.107	-14.860^***^		-.310	-.109	-15.021^***^
BMI (kg/m2)		.546	.085	12.877^***^		.366	.078	11.826^***^		.309	.076	11.503^***^
Parity	1	(ref.)				(ref.)				(ref.)		
	2	2.117	.055	7.578^***^		.905	.032	4.438^***^		.690	.029	3.962^***^
	3	2.553	.025	3.650^***^		1.316	.018	2.576^*^		1.367	.021	3.135^**^
	≥ 4											
Education	≤ 12	(ref.)				(ref.)				(ref.)		
	> 12	2.768	.058	8.743^***^		3.141	.090	13.586^***^		2.391	.080	12.116^***^
GWG										.046	.019	2.758^**^
NW		-1.199	-.034	-5.203^***^		-.380	-.015	-2.258^*^		-.436	-.020	-2.964^**^
NOF	1	(ref.)				(ref.)				(ref.)		
	≥ 2											
DM	CS	(ref.)				(ref.)				(ref.)		
	NIVD	-10.639	-.292	-40.137^***^		-8.169	-.305	-42.202^***^		-7.445	-.324	-45.060^***^
	EP	-12.060	-.216	-30.245^***^		-9.164	-.224	-31.470^***^		-8.385	-.239	-33.729^***^
	FD	-20.159	-.176	-26.474^***^		-15.238	-.182	-27.403^***^		-13.342	-.185	-28.113^***^
*F*		281.255^***^				304.462^***^				304.302^***^		
*R*^2^		0.106				0.114				0.125		
adj*R*^2^		0.106				0.114				0.125		

*BMI* body mass index, *GWG* gestational weight gain, *NW* neonatal weight, *NOF* number of fetus, *DM* delivery mode, *CS* cesarean section, *NIVD* non-instrumental vaginal delivery, *EP* episiotomy, *FD* forceps delivery, *B* unstandardized beta, *β* standardized beta, *t t* test statistic

^*^p < .05

^**^p < .01

^***^p < .001

## Discussion

Assessment of the PFMs is the basis for the prevention of PFDs. Pelvic floor surface EMG is a noninvasive technique that collects muscle motor potentials through surface electrodes, and is considered an effective method to assess the strength of the PFMs [25–27]. Previous studies have reported the association of EMG with UI and it is reliable and consistently predictive of clinical status variables [26, 28]. Surface EMG is widely used in China for the evaluation of PFM function because of its easy accessibility and cost-effectiveness and it has been considered effective to assess the function of the PFMs according to Branch of Women’s Health Care, Chinese Preventive Medicine Association [22]. Therefore, our study may contribute to predicting changes in pelvic floor muscle strength as well as its influencing factors to prevent pelvic floor muscle relaxation in the early stage.

Some sociodemographic characteristics may have an effect on PFM strength. Some studies have reported that aging may lead to a decrease in mechanical strength and predispose an individual to prolapse, UI and sexual dysfunction [29–32]. Likewise, in our study, the PFM rapid, tonic, and endurance contraction amplitudes all decreased when age increased.

BMI is also closely associated with PFDs. It has been reported that high BMI is a risk factor for PFDs, but it has also been reported that low BMI can also lead to pelvic organ prolapse (POP) [33, 34]. Univariate analysis and linear regression found that BMI was positively correlated with PFM strength in this study. In addition, some studies have also reported that GWG increased the subsequent risk of PFDs [35, 36]. In this paper, GWG contributed to the amplitude of endurance contractions after delivery. Both the increased BMI and GWG might result in increased intra-abdominal pressure [37]. As a result, the strength of the PFMs increased to sustain the increasing intra-abdominal pressure and visceral weight, similar to the correlation between BMI and muscle strength, and the changes might continue into the postpartum period [38–40].

The literature on the association between parity and the risk of PFDs indicates that multiparas are more likely to develop PFDs [41–43]. Unlike these outcomes, we found that PFM rapid, tonic, and endurance contraction amplitudes in secundiparas and tertiparas were higher than those in primiparas. This was an interesting outcome and might be an inspiration for us to rethink the effect of parity on PFM strength. Some ultrasound-based studies have found that injury to and structural deformation of the pelvic floor are independent of parity, suggesting that parity does not affect the pelvic floor as we believe [44, 45]. In addition, another study showed that the risk of levator avulsions, symptoms of POP, and clinical findings of POP were the same between primiparas and secundiparas, yet the occurrence of symptoms of POP increased for participants with three or more deliveries when compared to participants with one delivery [46]. Additionally, since sex education was not widespread in China teenagers, multiparas were more likely to receive sex education and Kegel training than primiparas, thus improving PFM strength [47]. Unfortunately, we did not collect information on whether they had received Kegel training.

Some studies have shown a significant relationship between educational level and PFM strength [42, 48]. Likewise, in the present study, PFM rapid, tonic, and endurance contraction amplitudes increased as the educational level increased. This result suggests that education increases women’s awareness about PFM strength.

As NW increases, the possibility of PFDs also increases. Previous studies have shown that excessive NW might harm PFM strength and was an independent risk factor for PFDs [49, 50]. Stepwise regression analysis showed that NW rather than the NOF, had a negative effect on PFM strength, including rapid, tonic, and endurance contractions, which was contrary to the results of univariate analysis. Women who had a baby that weight more than 4 kg or had twins or triplets were more likely to choose CS delivery, which has been confirmed to be a protective factor for PFM strength [51, 52]. A total of 34.1% participants with baby < 4 kg chose CS delivery, 66.8% of the participants with baby ≥ 4 kg chose CS delivery, 34.8% of the participants with a single baby chose CS delivery, and 91.6% of the participants with twins or triplets chose CS delivery (not shown in the tables).

Previous studies reported that vaginal delivery increased the risk of PFM dysfunction compared with cesarean delivery [53, 54]. Lima CTS et al. and JordiCassadó Garriga et al. found that EP and FD were associated with an increased risk of levator avulsion [55, 56]. Similarly, we found that the PFM rapid, tonic, and endurance contraction amplitudes in women with NIVD, EP, and FD were all lower than those in CS. Women with EP and FD showed a negative effect on PFM contraction capacity compared with women with NIVD.

## Conclusion

In this study, we found that age, NW, NIVD, EP, and FD were risk factors for PFM strength. Although BMI, parity (≤ 3) and GWG had a positive relationship with PFM strength, this is likely due to body’s adaptation ability and self-repair ability, rather than the benefits of weight gain or parity.

### Limitations

Only female participants were included; the number of multiparas (≥ 3) was too small to observe the changes in PFM strength when parity continued to grow, the assessment of surface EMG alone cannot reflect the overall function of the pelvic floor, and we lacked assessments of pelvic floor associated scales to assess the participants’ clinical symptoms, which made it difficult to relate our results to the clinic.

## Data Availability

The datasets analysed during the current study are not publicly available but are available from the corresponding author on reasonable request.
